# Management of T-Cell Lymphoma: In Quest of the Holy Grail

**DOI:** 10.3390/cancers13122919

**Published:** 2021-06-11

**Authors:** Sarah Péricart, Frédéric Escudié, David Grand, Pierre Brousset

**Affiliations:** Department of Pathology, IUCT-Oncopole, and Laboratoire d’Excellence TOUCAN, 1 Avenue Irène Joliot-Curie, CEDEX, 31059 Toulouse, France; pericart.sarah@iuct-oncopole.fr (S.P.); escudie.frederic@iuct-oncopole.fr (F.E.); grand.david@iuct-oncopole.fr (D.G.)

T-cell lymphomas (TCL) represent a very heterogeneous group of lymphoid tumors which are clearly distinct from B-cell neoplasms [1].

The first characteristic of TCL is their rarity (less than 10% of all types of lymphoma worldwide if we refer to the WHO classification of Tumors of Haematopoietic and Lymphoid Tissues [1] and the nationwide statistics from the French Lymphopath network [2]). The second particularity of these tumors is that their epidemiology displays significant racial and geographical variations, with subsets of tumors being over-represented in Asia [1,3]. The third particularity of TCL is the relatively high frequency of extra-nodal localizations with a specific emphasis on cutaneous forms. In the latter site, besides mycosis fungoides which represents the most frequent subtype, one observes many subgroups of rare tumors with primary cutaneous sites. The fourth particularity of TCL is their pathophysiology. Indeed, causative agents such as oncogenic viruses (EBV, HTLV1) are frequently at play in Asiatic cases [1,3]. In addition, the molecular mechanisms that drive T-cell oncogenicity are strikingly different from that of B-cell lymphomas. Recombination errors (immunoglobulin VDJ segment-based) are very frequent in B-cell lymphoma, leading to enhancer exchange mechanisms of gene activation, but are extremely rare in T-cell neoplasms, at least in mature ones. In the latter, kinase activation is more frequent, either obtained through the creation of fusion genes (X-ALK, SYK-ITK) or activating mutations (JAK-STAT) [1,3]. Other types of mutation that distinguish TCL occur in genes that are almost never mutated in B-cell tumors (*TET2*, *IDH2*, *DNMT3A* and *RHOA*, *FYN*–*TRAF3IP2* fusion) [1,3]. The fifth difference between B and T-cell lymphomas is the biology of the tumor microenvironment (TME). The signaling pathways that promote tumor growth seem to involve pivotal cells and specific cytokine networks in the TME. For example, one notices a critical role of follicular dendritic cells in the development of angioimmunoblastic T-cell lymphoma, in which the CXCL13-CXCR5 axis seems to be relevant. In addition, the presence of frequent EBV-positive B-immunoblasts in the TME of TCL argues in favor of both local and general immunosuppression. The presence of EBV+ B-cells expressing a latency of 2 or 3 indicates a significant decrease of cellular immune response. Indeed, LMP1+ EBV-infected B-cells are not allowed to proliferate in immunocompetent patients, in which they are immediately eliminated. The sixth distinct characteristic of systemic TCL compared to systemic B-cell lymphomas is their poor response to standard chemotherapy [3]. T-cell tumors display a very grim prognosis and so far remain inaccessible to efficient immunotherapy. In other words, there is no equivalent of anti-CD20 antibodies (Rituximab, Mabthera) in TCL therapies. Eliciting cellular immune response against malignant T-cells is highly challenging since the effector (helper/cytotoxic) cells are also diseased. Therapeutic advances are weak and only a few new drugs (anti-CD30 antibodies, anti-folate agents, histone deacetylase inhibitors, etc.) have been used with little or poor impact on patients’ survival [3]. Targeted therapies against mutated gene products (JAK1&2, SYK, PI3K...) represent an encouraging direction, with a few trials reporting some improvement in the overall survival in subsets of patients [3]. There are some trials using CART-cells, but the major difficulty is to circumvent a “fratricide” effect and a negative impact on normal T-cells [3]. In a recent report, Paul et al. [4] described an approach to target malignant T-cells through T-cell receptor (TCR) antigens. It is based on the principle that each normal or lymphoma T-cell expresses a unique TCR β chain generated from 1 of 30 TCR β chain variable (TCRBV) gene families [4]. They hypothesized that bispecific antibodies targeting a single TCRBV family member expressed in malignant T-cells could promote killing of these cancer cells, while preserving healthy T-cells that express other TCRBV family members [4]. Indeed, such a selective targeting is theoretically able to spare healthy T-cells and thus maintain cellular immune response. This strategy looks promising, but its efficacy must be confirmed in clinical models. 

Despite major progress in the understanding of molecular mechanisms involved in TCL, there is a large gap to translate the latter discoveries into clinical improvements. A better understanding and management of TCL will rely on major improvements of both diagnostic tools and data analysis. The latter are closely linked since new data permanently implement diagnostic tools. A better diagnosis will ensure optimal management towards personalized care. Achieving an accurate diagnosis in routine histopathology practice has become more challenging since the amount of tissue is decreasing due to the use of fine needle biopsies. Definite diagnosis can be reached less frequently with standard immunomorphological techniques and molecular analyses have become mandatory [2]. This implies a centralized review of difficult cases with easy access to NGS techniques [2]. Dedicated panels of genes can help take a definitive diagnostic decision and thus propose an accurate treatment. In this regard, liquid biopsies applied to subsets of tumors with informative gene mutations (*T-cell receptor genes*, *RHOA*, *TET2*, *IDH2*, *DNMT3A*, *JAK*-*STAT*, *NOTCH*, *ALK*.) look promising not only for diagnosis but also for treatment monitoring [3,5,6]. However, clues to significantly improve TCL treatments and increase patients’ survival are lacking. Indeed, NGS techniques have provided multiple mutations in genes involved in major signaling pathways [1,2,3]. The latter remain difficult to target and we know from other tumor subtypes (solid tumors) that targeted therapies are frequently associated with relapse. 

The research paradigm has to be changed dramatically to switch from a linear determinist approach to a more integrated analysis of complex data. There have been some attempts to move towards precision medicine in TCL management [7]. Functional biomarker-based strategies have been proposed for identification of the most efficacious drugs or combinations for targeted therapies in TCL, but such approaches present important limitations because many key biological parameters of the tumor complexity are dismissed [8,9]. Another direction is to perform data mining to ensure extraction of patterns and knowledge from large amounts of data. Data mining is frequently coupled with computer decision support systems, including artificial intelligence (AI). Comprehensive studies based on multi-omics (genomics, proteomics, metabolomics), imaging, patient clinical data (including those obtained from connected objects with specific apps of follow up) is the direction to take (Figure 1) [10,11]. Data mining and AI algorithms (machine learning) could be used to analyze huge amounts of complex and heterogeneous data sets [10,11]. From these analyses based on data integration, researchers and physicians will have access to holistic disease molecular mechanisms [10]. This approach will allow identification of diagnostic and prognostic markers, and the monitoring of patient outcomes. Lastly, the routine access to constitutional whole genome for all patients will open new doors for truly personalized (idiosyncratic) medicine [11]. 

## Figures and Tables

**Figure 1:**
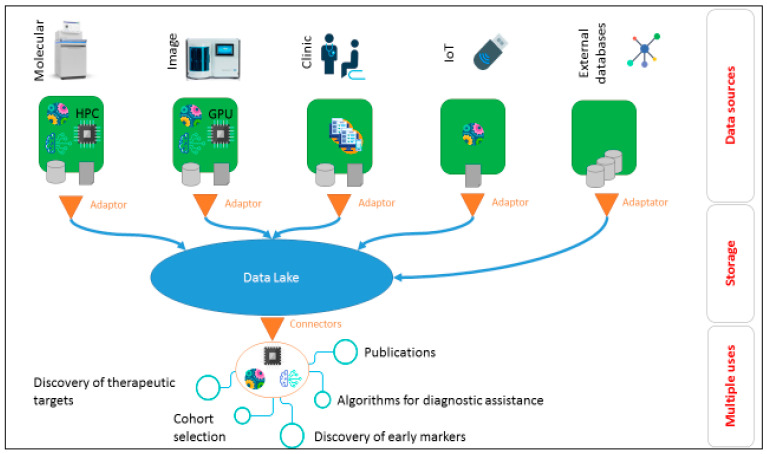
Precision medicine and strategies of data hybridization. IOT: internet of things; GPU: graphic processing unit; HPC: high performance computing. Icons from www.flaticon.com (accessed on 15 May 2020).

## Abbreviations

TCL: T-cell lymphomas, TME: tumor microenvironment, EBV: Epstein-Barr virus, HTLV1: human T-cell leukemia/lymphoma virus 1.
